# Governance and Capacity to Manage Resilience of Health Systems: Towards a New Conceptual Framework

**DOI:** 10.15171/ijhpm.2017.36

**Published:** 2017-04-04

**Authors:** Karl Blanchet, Sara L. Nam, Ben Ramalingam, Francisco Pozo-Martin

**Affiliations:** ^1^Department of Global Health and Development, Faculty of Public Health and Policy, London School of Hygiene & Tropical Medicine, London, UK.; ^2^Options Consultancy Services Ltd, London, UK.; ^3^Institute of Development Studies, Brighton, UK.

**Keywords:** Resilience, Health Systems, Governance, Management, Complexity

## Abstract

The term resilience has dominated the discourse among health systems researchers since 2014 and the onset of the Ebola outbreak in West Africa. There is wide consensus that the global community has to help build more resilient health systems. But do we really know what resilience means, and do we all have the same vision of resilience? The present paper presents a new conceptual framework on governance of resilience based on systems thinking and complexity theories. In this paper, we see resilience of a health system as its capacity to absorb, adapt and transform when exposed to a shock such as a pandemic, natural disaster or armed conflict and still retain the same control over its structure and functions.

## Introduction

The term resilience has dominated the discourse among health systems researchers since 2014 and the onset of the Ebola outbreak in West Africa.^1,2^ There is wide consensus that the global community has to help build more resilient health systems. But do we really know what resilience means, and do we all have the same vision of resilience? The emergence of the term ‘resilience’ for some echoes the 1990s experience with ‘sustainability’ in that it seems to have the function of accommodating various political and scientific paradigms.^3^ Resilience is seen as a ‘boundary term’^4^ that is at the crossroad between politics and science.^5^ As such, it may have the function of building political consensus and aligning and enabling the co-existence of several different agendas,^6^ which may explain why the term resilience may remain contested and ambiguous. However, it remains important for health systems researchers and practitioners to clarify the meaning of the concept and have common guidance as to its use.

Strengthening the capacity of health systems to manage resilience is critical to effectively continue delivering essential preventative and curative healthcare services to populations. This requires adapting and transforming the structure and properties of the health system to move it away from undesirable risk situations.^7^ However, how do we recognize situations of risks? How do we know what properties of the system are better adapted to certain circumstances? What are the potential effects of alternative routes? Who makes decisions on the directions of the health system? These are critical questions on the management and governance of resilience on how to manage the capacities of health systems.

Our objective through this paper is to value the various perspectives and maintain diversity of opinions while providing a common framework to help researchers dialog with each other and generate more studies in this field. The present paper presents a new conceptual framework for research based on systems thinking and complexity theories.^8-10^

## Towards a New Conceptual Framework on Governance of Resilience

Governance relates to the implicit and explicit rules and institutions that shape power, relationships between actors, and the actions of these actors. Managing resilience of health system resides in the capacity of managing actors, networks and institutions that have an influence on the health system.^11^ With the emergence of system thinking and complexity science, the world is now described as a network of systems interacting between each other and influencing different levels of society.^12,13^ Dynamic systems of different sizes interact across multiple scales^14-17^ and affect systems’ characteristics in relation to the nature, frequency and intensity of shocks experienced.^18^ Drawing on the resilience literature,^19,20^ it is important to define both what resilience is, in specific contexts, and explain how it can be managed in those contexts. In this paper, we see resilience of a health system as its capacity to absorb, adapt and transform when exposed to a shock such as a pandemic, natural disaster, armed conflict or a financial crisis and still retain the same control over its structure and functions (adapted from^11,21,22^).

We propose a new conceptual framework adapted from environmental studies^11^ to help analyse the governance of health systems resilience. We developed this framework based on a scoping review of the latest definitions of resilience of systems in the health sector and elsewhere (eg, ecology and urban studies). Based on discussions with experts, a new conceptual framework needs to be operational (ie, to be able to be translated into measurable indicators to be tested) and comprehensive, defined by Sabatier^23^ as being able to take into account “conflicting values and interests, information flows, institutional arrangements and variation in the socioeconomic environment.”

**Figure 1:**
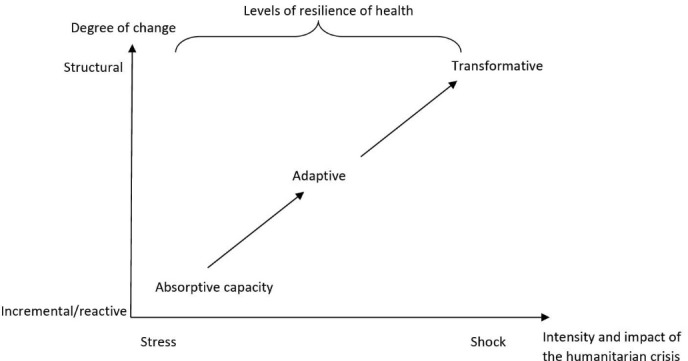
The Levels of Resilience of Health Systems by Intensity of the Humanitarian Crisis and Degree of Change (adapted from OCHA^24^).

With a firm grounding in complex systems sciences, the management of resilience of health systems is characterised by four main dimensions interlinked with each other: (*i*) capacity to collect, integrate and analyse different forms of *knowledge* and information; (*ii*) ability to anticipate and cope with *uncertainties* and surprises; (*iii*) capacity to manage *interdependence*: to engage effectively with and handle multiple- and cross-scale dynamics and feedbacks; and finally (*iv*) capacity to build or develop *legitimate* institutions that are socially accepted and contextually adapted (see Figure 1). The potential value of this framework is that it integrates all the different approaches to resilience alluded to in health systems thinking – the building blocks, the systemic properties, and the enabling institutional environment – into one single approach for use by researchers, practitioners and policy-makers, and each dimension is described here.

**Figure 2:**
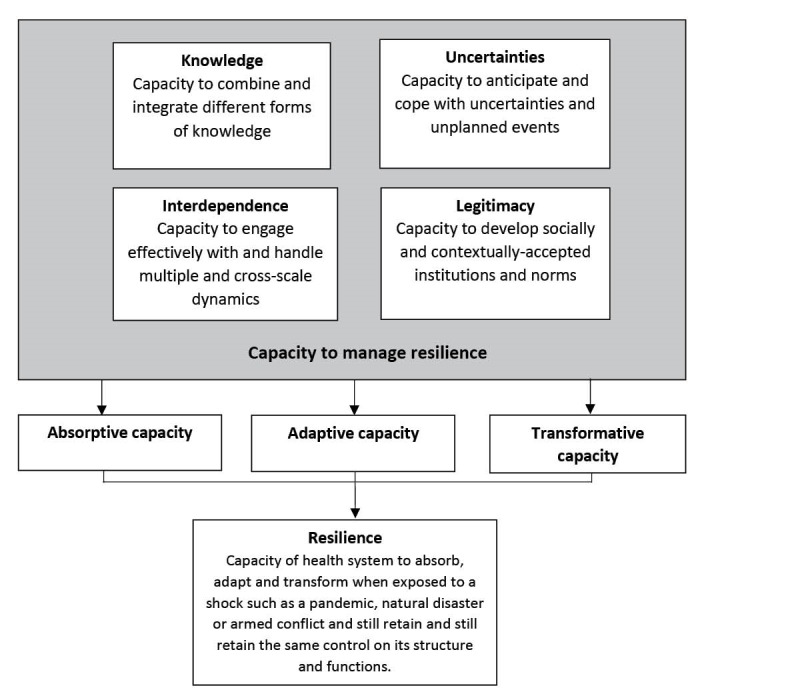
A Conceptual Framework: The Dimensions of Resilience Governance (adapted from Lebel et al^11^).

Based on frameworks used in ecology, three levels of resilience can be applied to health systems: absorptive capacity, adaptive capacity and transformative capacity (see Figure 2).

Within health systems thinking, the *absorptive capacity* relates to the capacity of a health system to continue to deliver the same level (quantity, quality and equity) of basic healthcare services and protection to populations despite the shock using the same level of resources and capacities.^25^ *Adaptive capacity* is the capacity of the health system actors to deliver the same level of healthcare services with fewer and/or different resources, which requires making organisational adaptations.^22,26-28^ Finally, the *transformative capacity* describes the ability of health system actors to transform the functions and structure of the health system to respond to a changing environment.^11,22,29,30^ We propose a new conceptual framework to define the four dimensions that need to be taken into account in terms of governance of resilient health systems.

## Capacity to Combine and Integrate Different Forms of Knowledge

Anticipating shocks and events such as public health outbreaks requires a functional disease surveillance system (in the case of outbreaks) to inform health services managers on the occurrence of pandemics and the state of transmission of the disease.^31^ However, the nature of the knowledge that needs to be collected and processed to ensure and measure resilience needs to extend beyond the sphere of the health system.^32,33^ For example, health systems planners need to understand the current resources available, where existing gaps in resources exist or where weaknesses in the health system lie as well as the current health status of the population and their health priorities. Furthermore, they need to be able to monitor risks and threats that may lie beyond the direct realm of the health system and may, for example, be related to the economic sphere or the political context.^34,35^

Having access to such different types of knowledge is related to the capacity to engage with a diversity of actors belonging to differing spheres of society. This has been extensively documented in social network analysis, exemplified with the notion of social brokers, ie, individuals who create links between users and researchers.^36^ The social brokers in a health system help coordinate actors in times of crises or shocks and build bridges between different groups within the system and beyond it.^37-40^ Thus, identifying actors who are social brokers and giving credence to their role in combining and integrating different forms of knowledge will support strengthening resilience of a health system.

## Uncertainties: Capacity to Anticipate and Cope With Uncertainties and Unplanned Events

Resilience can be understood in terms of the adaptability of health systems.^9,19,27,40^ Adaptability is the capacity of the actors in a system to respond to stresses and shocks.^41^ The adaptability of systems is mainly a function of the actions and decisions taken by individuals, networks and groups managing these systems.^12,42^ For example, using complex adaptive systems analysis, MacKenzie et al^43^ showed that the capacity of the health system in Northern Nigeria to adapt to an outbreak required not only a capacity to cover all six building blocks of the health system, but also required access to flexible, adaptable resources to respond to unexpected shocks, such as outbreaks, as opposed to rigid aid or typically inflexible government funding.

Even when planners have relevant knowledge and flexible systems to plan for uncertainties or react to shocks, decision-making for individuals is complex. Complexity science reveals challenges to rapid and appropriate decision making, where, for example, decisions made by actors may not respond to the needs as identified by access to relevant and different forms of knowledge. Scholars have found that there is a relationship between the structure of networks, the type of links between actors (ie, the degree of bonding between actors of the system or bridging links with other systems) and the resilience of social-ecological systems.^38,39^ Decision-makers may react to knowledge in response to their ‘survival instincts’^44^ over the needs of the health system or may be inappropriately influenced by individuals’ interests and the opinions of peers who are part of the same social network. Adopting social network analysis may facilitate understanding of how actors within a system and with other systems are linked and can inform or enable mitigation of delays in responses.

## Interdependence: Capacity to Engage Effectively With and Handle Multiple and Cross-Scale Dynamics

Recognising that health systems are embedded within other complex structures (eg, political, economic, judiciary, social and ecological systems) and across scales (local, sub-national, national and international levels) alludes to how health systems are affected by factors, which may not seem to be directly linked to public health, as mentioned above.^22,45^ In the policy context, this was described by Blanchet et al^9^ who showed that the structure of the physical rehabilitation system in Somaliland was transformed following changes in national security and international donors’ strategies. The degree to which health systems are influenced by non-health systems is often all too apparent when health systems are not resilient.^46^ For example, the inadequate capacity of fragile health systems underpinned the challenges in responding to the Ebola outbreak, in countries afflicted by decades of conflict, weak economies, and entrenched poverty.^2^ Building resilience in the wake of Ebola will need to take all of these factors into account: not treating the crisis solely as a medical emergency, but as a profound and long-term failure of economic and social development.^13^

Social network analysis has illustrated how *social brokers* support resilience in the capacity of the health system to engage with a diversity of actors belonging to these other socio-political structures and spheres. The social brokers in a health system help coordinate actors in times of crises or shocks and build bridges between different groups within the system.^37-40^ Identifying who the social brokers are can enable application of their skills as brokers in support of strengthening resilience by engaging them in multiple and cross-scale dynamics.

## Legitimacy: Capacity to Develop Socially and Contextually-Accepted Institutions and Norms

Another important component of resilient health systems arising in the literature relates to the necessity of community trust and ownership. This can be built through an inclusive consultation process engaging communities meaningfully as the users of the health system in the development of policies and management of healthcare services where patients are placed at the centre of the system.^47,48^ Importantly, person-centred management of health systems needs to happen at every level.^1^ Kieny et al^49^ showed that responding to the Ebola outbreak requires trust and accountability to exist or be built at every level of the health system: from the patient, to the community health worker, the nurses at the health centre, to medical and managerial staff at higher level. The Ebola outbreak has demonstrated the importance of building a trusting relationship with populations: to mitigate the situation where communities avoid using health facilities by fear of contamination.^50^ The violence against healthcare workers also showed the disconnect between communities and health services.^51^ To achieve this, the existence of client-based information systems on quality of care for each facility is needed, such as that initiated by the Ministry of Health and Sanitation in Sierra Leone through the scorecards developed for the Facility Improvement Team (FIT) Assessment initiative, for example, where clients have access to transparent information about quality delivered by each facility.^52,53^

## Conclusion

In this paper we put forward a new framework for the analysis of health systems resilience. This framework extends previously existing frameworks from ecological science to the study of health systems. Resilience is defined here as the capacity of a health system to absorb, adapt and transform when exposed to a shock and still retain control over its structure and functions. Thus, health systems are resilient if they exhibit absorptive, adaptive or transformational capacity in the face of shocks of different intensity. In our framework, we bring new elements of resilience governance by defining four key dimensions to manage health system resilience. The four dimensions consist in understanding: (*i*) the mechanisms through which health system actors collect, systematise and interpret complex information, as well as the way this information feeds into complex decision-making processes; (*ii*) the strategies health system actors may use to manage uncertainty and surprises; (*iii*) the interdependence of health systems with other complex systems; and (*iv*) the approaches through which health systems develop socially- and contextually- acceptable institutions and norms. This framework can be used by health systems researchers, health practitioners and policy-makers to explore the characteristics of resilient health systems and put forward context-specific, evidence-based and comprehensive approaches to improve resilience. Ultimately, the resilience of health systems is of great importance to underpin the health resilience of poor and vulnerable populations as well as the capacity of the systems to respond to peoples’ changing – and unpredictable – needs.

## Acknowledgments

We thank Dr. Susannah Mayhew and Dr. Sandra Mounier-Jack for their feedback and input.

## Ethical issues

No ethical clearance was required as this is a conceptual paper.

## Competing interests

Authors declare that they have no competing interests.

## Authors’ contributions

KB wrote the first draft of the paper. All co-authors contributed important revisions to the paper. All co-authors approved the final submitted version of the paper.

## Authors’ affiliations

^1^Department of Global Health and Development, Faculty of Public Health and Policy, London School of Hygiene & Tropical Medicine, London, UK. ^2^Options Consultancy Services Ltd, London, UK. ^3^Institute of Development Studies, Brighton, UK.
